# A set of novel SNP loci for differentiating continental populations and three Chinese populations

**DOI:** 10.7717/peerj.6508

**Published:** 2019-03-29

**Authors:** Xiao-Ye Jin, Yuan-Yuan Wei, Qiong Lan, Wei Cui, Chong Chen, Yu-Xin Guo, Ya-Ting Fang, Bo-Feng Zhu

**Affiliations:** 1 Key laboratory of Shaanxi Province for Craniofacial Precision Medicine Research, College of Stomatology, Xi’an Jiaotong University, Xi’an, China; 2 Clinical Research Center of Shaanxi Province for Dental and Maxillofacial Diseases, College of Stomatology, Xi’an Jiaotong University, Xi’an, China; 3 College of Medicine and Forensics, Xi’an Jiaotong University Health Science Center, Xi’an, China; 4 Department of Forensic Genetics, School of Forensic Medicine, Southern Medical University, Guangzhou, China

**Keywords:** Ancestry informative markers, Biogeographical origins, SNP, Chinese populations, Continental populations

## Abstract

In recent years, forensic geneticists have begun to develop some ancestry informative marker (AIM) panels for ancestry analysis of regional populations. In this study, we chose 48 single nucleotide polymorphisms (SNPs) from SPSmart database to infer ancestry origins of continental populations and Chinese subpopulations. Based on the genetic data of four continental populations (African, American, East Asian and European) from the CEPH-HGDP database, the power of these SNPs for differentiating continental populations was assessed. Population genetic structure revealed that distinct ancestry components among these continental populations could be discerned by these SNPs. Another novel population set from 1000 Genomes Phase 3 was treated as testing populations to further validate the efficiency of the selected SNPs. Twenty-two populations from CEPH-HGDP database were classified into three known populations (African, East Asian, and European) based on their biogeographical regions. Principal component analysis and Bayes analysis of testing populations and three known populations indicated these testing populations could be correctly assigned to their corresponding biogeographical origins. For three Chinese populations (Han, Mongolian, and Uygur), multinomial logistic regression analyses indicated that these 48 SNPs could be used to estimate ancestry origins of these populations. Therefore, these SNPs possessed the promising potency in ancestry analysis among continental populations and some Chinese populations, and they could be used in population genetics and forensic research.

## Introduction

Ancestry informative markers, which demonstrated distinct allele frequency differences among different populations (Frudakis et al., 2003; Shriver et al., 1997), could be used to infer biogeographical origins of unknown biological samples and discern population substructure. They are also conductive to forensic investigations by providing investigative clues about the biogeographical origins of the unknown suspect. To date, a great many AIM panels for different purposes have been developed (Li et al., 2016; Phillips, 2015; Phillips et al., 2013). It is noteworthy that AIM panels differentiating different continental populations might not be appropriate to differentiate populations in the same continent, and these panels could even produce prediction errors for ancestry inferences of these populations. The two-tier AIM panels were recommended for this issue: one for estimating ancestry origins of major continental populations and the other for populations in the same continent (Kidd et al., 2014). Besides, Pakstis et al. (2017) stated that the identification of highly differentiated SNPs in relatively neglected geographical regions or subpopulations from the same continent should be encouraged because these markers could further improve and fine-tune the extant SNP panels.

China, one of the world’s earliest civilizations, consists of 56 officially identified ethnic groups. Previous studies found extensive genetic variations among Han populations and minority groups in China (Wang et al., 2018; Zhang et al., 2015; Zhao & Lee, 1989; Zhao et al., 1991). Some researchers selected some markers to differentiate Chinese populations. For examples, Qin et al. (2014) provided 150 SNPs for ancestry analysis of northern Han and southern Han; Sun et al. (2016) presented twelve multi-InDels to differentiate Han and Tibetan populations. Ancestry analysis among more populations in China should be conducted to provide more valuable information for genome-wide association study and forensic investigation. As is known to us, Han people are the largest ethnic group in the world and distribute in many regions. Uygur individuals mainly live in the Xinjiang Uygur Autonomous Region where extensive population movements occurred in history and they possess admixture genetic components of East Asians and Europeans (Xu et al., 2008). Mongolian group is one of fifty-six ethnic groups in China. During the period of the Yuan dynasty, the governor of China and his soldiers began their expedition to Europe which might lead to some Mongolian individuals dispersing in different regions (https://en.wikipedia.org/wiki/Mongols). Although genetic differentiations among Han, Uygur and Mongolian populations existed (Zhang et al., 2015), no research on ancestry analyses of these populations was conducted.

In this study, based on population data assembled in CEPH-HGDP (Li et al., 2008), a great number of SNPs were selected for distinguishing four major regions (Africa, America, Europe and East Asia). Among these SNPs, the SNPs with high differentiations among Han, Uygur and Mongolian populations were further selected to infer ancestry origins of these populations.

**Table 1:** Detailed information of populations used in this study and their corresponding sample sizes.

Datasets	Populations	Abbreviations	Continents	Sources	Sample sizes
Training set	Biaka Pygmy	–	Africa	CEPH-HGDP	22
	Mbuti Pygmy	–	Africa	CEPH-HGDP	13
	Bantu^a^	–	Africa	CEPH-HGDP	19
	Yoruba	–	Africa	CEPH-HGDP	21
	Mandenka	–	Africa	CEPH-HGDP	22
	Brazian^b^	–	America	CEPH-HGDP	22
	Maya	–	America	CEPH-HGDP	21
	Pima	–	America	CEPH-HGDP	14
	Basque	–	Europe	CEPH-HGDP	24
	French	–	Europe	CEPH-HGDP	28
	Italian^c^	–	Europe	CEPH-HGDP	49
	Orcadian	–	Europe	CEPH-HGDP	15
	Adygei	–	Europe	CEPH-HGDP	17
	Russian	–	Europe	CEPH-HGDP	25
	Cambodian	–	East Asia	CEPH-HGDP	10
	Dai	–	East Asia	CEPH-HGDP	10
	Han	–	East Asia	CEPH-HGDP	44
	Miao	–	East Asia	CEPH-HGDP	10
	Mongolian	–	East Asia	CEPH-HGDP	10
	She	–	East Asia	CEPH-HGDP	10
	Tu	–	East Asia	CEPH-HGDP	10
	Tujia	–	East Asia	CEPH-HGDP	10
	Yi	–	East Asia	CEPH-HGDP	10
	Japanese	–	East Asia	CEPH-HGDP	28
	Yakut	–	East Asia	CEPH-HGDP	25
Testing set	Esan in Nigeria	ESN	Africa	1000 Genomes Phase 3	99
	Yoruba in Ibadan, Nigeria	YRI	Africa	1000 Genomes Phase 3	108
	Finnish in Finland	FIN	Europe	1000 Genomes Phase 3	99
	British in England and Scotland	GBR	Europe	1000 Genomes Phase 3	91
	Han Chinese in Bejing, China	CHB	East Asia	1000 Genomes Phase 3	103
	Japanese in Tokyo, Japan	JPT	East Asia	1000 Genomes Phase 3	104
Three subpopulations in China	Uygur	–	Central Asia	CEPH-HGDP	10
	Han	–	East Asia	CEPH-HGDP	44
	Mongolian	–	East Asia	CEPH-HGDP	10

**Notes.**

a Bantu population includes Kenya Bantu and South African Bantu populations.

b Brazian population includes Karitiana and Surui populations.

c Italian population includes Sardinian, Tuscan and Bergamo populations.

## Material and Methods

### Reference populations

Twenty-five populations within four major geographical regions were chosen as the training set for preliminary assessments of selected SNPs. Six testing populations in three continents included Esan, Yoruba, Finnish, British, Beijing Han and Japanese populations, of which genetic data were downloaded from 1000 Genomes Phase 3 (Genomes Project Consortium et al., 2015). Genetic data of three subpopulations in China (Han, Mongolian, and Uygur) was obtained from CEPH-HGDP (Li et al., 2008). Detailed descriptions of these populations and their corresponding sample sizes were given in Table 1. Besides, genetic data of 48 SNPs in training, testing and three Chinese populations were presented in Table S1.

### Criteria for SNP selection

SPSmart includes the genetic data of 1000 Genomes Phase I, HapMap release #28, Perlegen complete data set and the Stanford University and Michigan University CEPH-HGDP panels, which is developed to help researchers use and combine different datasets and do some statistical analyses of interest (Amigo et al., 2008). SNPs were chosen from SPSmart online tool when they met the following criteria: (1) SNP loci should locate in intron regions. (2) SNP loci were bi-allelic genetic markers; (3) SNP loci were located on different chromosomes or at least 10 Mb distances on the same chromosome; (4) Ancestral allele frequency differences between continental populations were at least 0.3; (5) SNP selected must conform to Hardy-Weinberg equilibrium (HWE) in all reference populations. Next, SNPs whose frequency differences among Han, Uygur and Mongolian populations were more than 0.3 were used for further analysis. Besides, we also retained some SNP loci that showed high genetic differentiations among continental populations/three Chinese populations. Finally, forty-eight SNPs were selected to differentiate continental populations and three Chinese populations. General information of 48 SNPs was given in Table 2.

### Statistical analysis

HWE tests of SNP loci in 25 training populations were estimated by Genepop software v4.0 (Rousset, 2008). Allele frequencies of 48 SNP loci in 25 training populations were calculated by PowerStats software v1.2 (Promega, Madison, WI, USA). The informativeness for assignment (*In*) values of 48 SNP loci in four continental populations (African, American, European, and East Asian) were calculated by Infocalc program v1.1 (Rosenberg et al., 2003) based on the genetic data of 25 training populations. Ancestral allele frequency heatmap and the boxplot of *In* values of 48 SNPs were plotted by R software v3.3 (R Core Team, 2016). Principal component analysis (PCA) of four continental populations including 25 training populations was conducted by PLINK software v1.9 (http://www.cog-genomics.org/plink/1.9/), and then scatter plot of these populations was plotted by R software v3.3. Population genetic structure of 25 training populations at *K* = 2–5 and cross-validation error of each *K* value were performed by ADMIXTURE software v1.3 (Alexander, Novembre & Lange, 2009). Graphical results of estimated ancestry proportions were conducted with the CLUMPAK online tool (Kopelman et al., 2015).

To further evaluate discrimination efficiencies of 48 SNPs for continental populations, ancestry components of six testing populations were estimated by ADMIXTURE software v1.3 and their results were shown in the form of beeswarm by R software v3.3. Next, twenty-two training populations (excluding from American populations) were treated as reference populations and six testing populations were blind samples. PCA and Naïve Bayes analysis of these populations including reference populations and six testing populations were conducted with PLINK software v1.9 and the Snipper App suite v2.5 (http://mathgene.usc.es/snipper), respectively.

**Table 2:** General information and ancestral allele frequencies of 48 SNP loci in different continental populations.

Rs numbers	Alleles^a^	Chromosomes^a^	Positions (bp)^a^	African^b^	American^b^	European^b^	East Asian^b^
rs10918196	C/T	1	165478920	0.8351	0.3947	0.6108	0.2429
rs2801178	G/T	1	14881716	0.8608	0.2368	0.7753	0.4774
rs4652825	A/G	1	183824189	0.7320	0.4737	0.8101	0.6215
rs10779958	A/C	2	74528651	0.3763	0.5000	0.8481	0.1921
rs1161474	C/T	2	239209361	0.7320	0.6053	0.3829	0.5424
rs7570426	A/C	2	3325638	0.5567	0.8947	0.9051	0.4915
rs11716005	C/T	3	18394010	0.9742	0.9474	0.8101	0.5621
rs301927	A/G	3	97627774	0.7423	0.8947	0.0918	0.6638
rs4533619	A/G	3	42248320	0.2835	0.3246	0.1456	0.7345
rs4894436	G/T	3	171401588	0.6649	0.0789	0.6551	0.0847
rs6446081	C/T	3	59723035	0.8866	0.8070	0.7532	0.5480
rs12650562	C/T	4	23799564	0.4897	0.7456	0.5032	0.5593
rs3762894	C/T	4	99144933	0.7268	1.0000	0.8291	0.3757
rs2400219	C/T	5	146472334	0.1546	0.2193	0.2057	0.7994
rs277329	A/C	5	54158354	0.8351	0.8158	0.8133	0.3814
rs35414	C/T	5	33969523	1.0000	0.8509	0.3956	0.8644
rs4704322	C/T	5	76526649	0.9639	0.5175	0.7880	0.1299
rs871722	A/G	5	17437385	0.8918	0.2368	0.1361	0.0876
rs1857859	A/G	6	100446711	0.8557	0.4123	0.7025	0.5678
rs4711760	C/T	6	44027931	0.7990	0.7018	0.3291	0.9379
rs947612	A/G	6	73028938	0.8608	0.7018	0.2500	0.7655
rs2373177	C/T	7	147717307	0.6031	0.6053	0.7152	0.7386
rs4646437	G/A	7	99767460	0.8505	0.2456	0.1108	0.1243
rs7795646	A/G	7	120683673	0.5773	0.7632	0.2057	0.4774
rs2595599	A/G	8	92646120	0.4124	0.3947	0.7962	0.2542
rs351554	A/C	8	16149886	0.7680	0.5439	0.1329	0.4972
rs4738110	A/C	8	71193716	0.9588	0.3509	0.7342	0.3136
rs10965206	A/G	9	229826	0.4845	0.5877	0.8766	0.5791
rs4743923	C/T	9	93488779	0.0361	0.3333	0.4019	0.4096
rs16917217	A/G	10	18370276	0.2938	0.2456	0.3513	0.6441
rs4933165	C/T	10	89903145	0.8557	0.5439	0.8956	0.2147
rs11218323	C/T	11	99080380	0.6856	0.8070	0.8734	0.5256
rs1470253	A/G	11	20099911	0.7732	0.2018	0.7437	0.1384
rs1032332	T/C	12	72568351	0.5825	0.2632	0.1361	0.5311
rs17650122	A/G	13	29010756	0.5052	0.5877	0.5696	0.3220
rs3782972	C/T	13	94450792	0.9330	0.2544	0.7468	0.3220
rs7325443	C/T	13	111238209	0.7938	0.3246	0.8070	0.3079
rs10148212	A/C	14	66628895	0.2917	0.5357	0.9209	0.5028
rs10852189	C/T	15	93163645	0.5158	0.2281	0.3228	0.5141
rs9806693	A/G	15	78892045	0.6495	0.5439	0.2437	0.6780
rs170359	A/G	16	57361752	0.6649	0.3333	0.0316	0.1412
rs7219900	C/T	17	15471179	0.7474	0.2105	0.1076	0.3475
rs11152349	A/G	18	62566413	0.7062	0.6316	0.7057	0.3023
rs528438	T/C	18	28093911	0.2165	0.7632	0.3038	0.8079
rs1205357	C/T	20	34289960	0.8711	0.0088	0.1076	0.2147
rs162315	A/G	20	57233824	0.2268	0.6667	0.7025	0.6864
rs2178832	G/A	21	35449600	0.2010	0.4737	0.5443	0.2288
rs361557	A/G	22	18128456	0.9330	0.7456	0.1835	0.5650

**Notes.**

a Information of each SNP locus is shown according to the report of dbSNP build 152.

b Ancestral allele frequencies of 48 SNPs in four continental populations are obtained based on the genetic data of 25 training populations in Table 1.

Ancestry analyses among Uygur, Han and Mongolian populations were conducted based on the 48 SNPs. First of all, allele frequencies of 48 SNPs in these three populations were calculated by PowerStats software v1.2. *In* values of 48 SNP loci in Uygur, Han and Mongolian populations were also calculated with Infocalc program v1.1. Secondly, PCA of these populations was also conducted by PLINK software v1.9. Ancestral allele frequency heatmap and the boxplot of *In* values for 48 SNPs, and scatter plot of these three populations were generated by R software v3.3 (R Core Team, 2016). Genetic structure analyses of these populations at *K* = 3 were conducted by ADMIXTURE software v1.3 and graphical results were plotted by CLUMPAK online tool. In the end, multinomial logistic regression analyses of these three populations were conducted by Snipper App suite v2.5.

## Results

### Frequency distributions and population specific *In* values of 48 SNPs

After applying Bonferroni correction (the significant level = 0.05/48 = 0.00104), the selected 48 SNPs all conformed to HWE in 25 training populations (Table S2).

**Figure 1:**
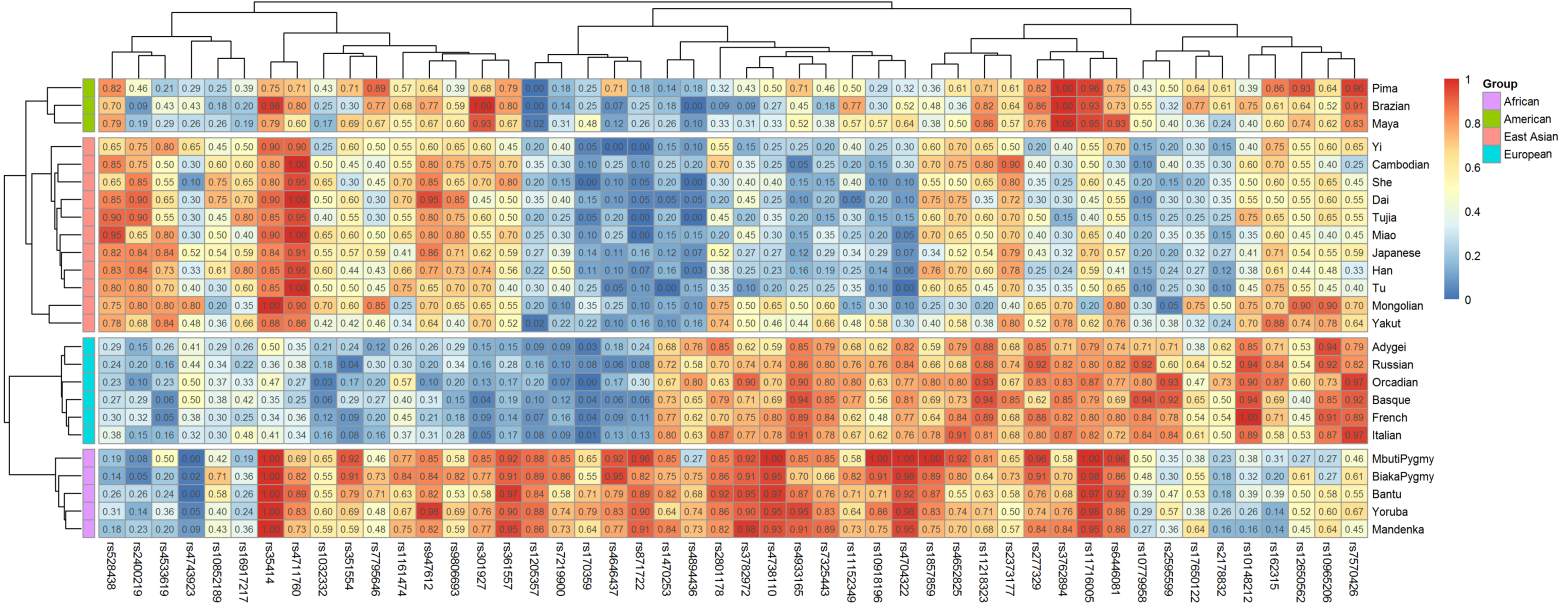
Ancestral allele frequency heatmap of 48 SNPs in 25 training populations from different continents. Different colors represent for different levels of frequency values: blue for low value, red for high value.

Ancestral allele frequencies of 48 SNP loci in 25 reference populations were shown in Fig. 1. Color contrasts of SNPs in pairwise populations reflected genetic differentiations of pairwise populations: more apparent color contrasts of pairwise populations were, larger genetic differentiations pairwise populations possessed, and vice versa. Besides, the SNPs with distinct color contrasts in pairwise continental populations contributed to differentiating these populations. For example, ancestral allele frequencies of the rs10918196 locus in African, American and East Asian populations were 0.8351, 0.3947 and 0.2429 (Table 2), implying the locus was beneficial to distinguish African populations from the other two continental populations. The phylogenetic tree above the heatmap (Fig. 1) revealed the relationships of SNPs: SNPs showed similar frequency distributions in continental populations tended to locate in the same sub-branches, and vice versa. The phylogenetic tree in the left part of the graph reflected genetic divergences of different continental populations: populations with the same biogeographical origins located in the same sub-branches.

**Figure 2:**
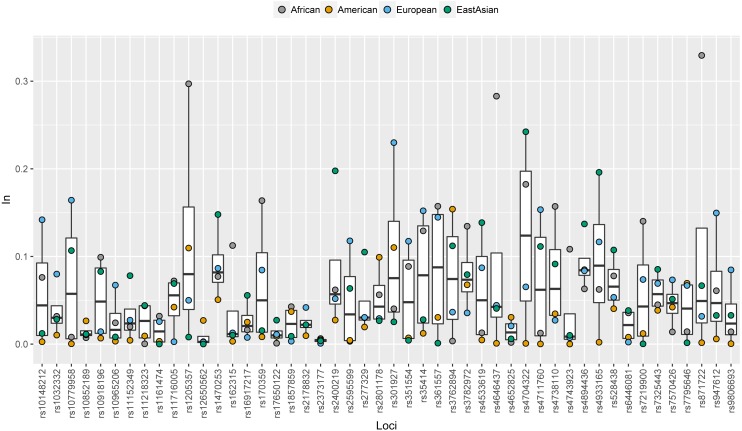
Population specific *In* values of 48 SNPs in African, American, European and East Asian populations.

*In* values of 48 SNPs in African, American, European and East Asian populations were shown in Fig. 2 and Table S3. Similar with allele frequency differences, *In* values were also used to evaluate the degree of population differentiations: genetic markers with high *In* values in the certain population contributed to differentiating the population from the other populations (Phillips, 2015). For these 48 SNPs, there were 12, 11, 10, and three SNPs with relatively high *In* values (≥0.1) in African, East Asian, European, and American populations, respectively. Besides, some SNPs which had similar frequency distributions among different continental populations showed low *In* values in these four continental populations (Fig. 2). Besides, we also calculated the cumulative *In* values of 48 SNPs in African, European, East Asian, and American populations, which were 3.4312, 3.0343, 2.7727 and 1.3028, respectively (Table S3). The low cumulative *In* values in American populations indicated these SNPs might possess lower power to differentiate American populations from the other populations.

### PCA and ancestral component analysis of 25 training populations based on 48 SNPs

**Figure 3:**
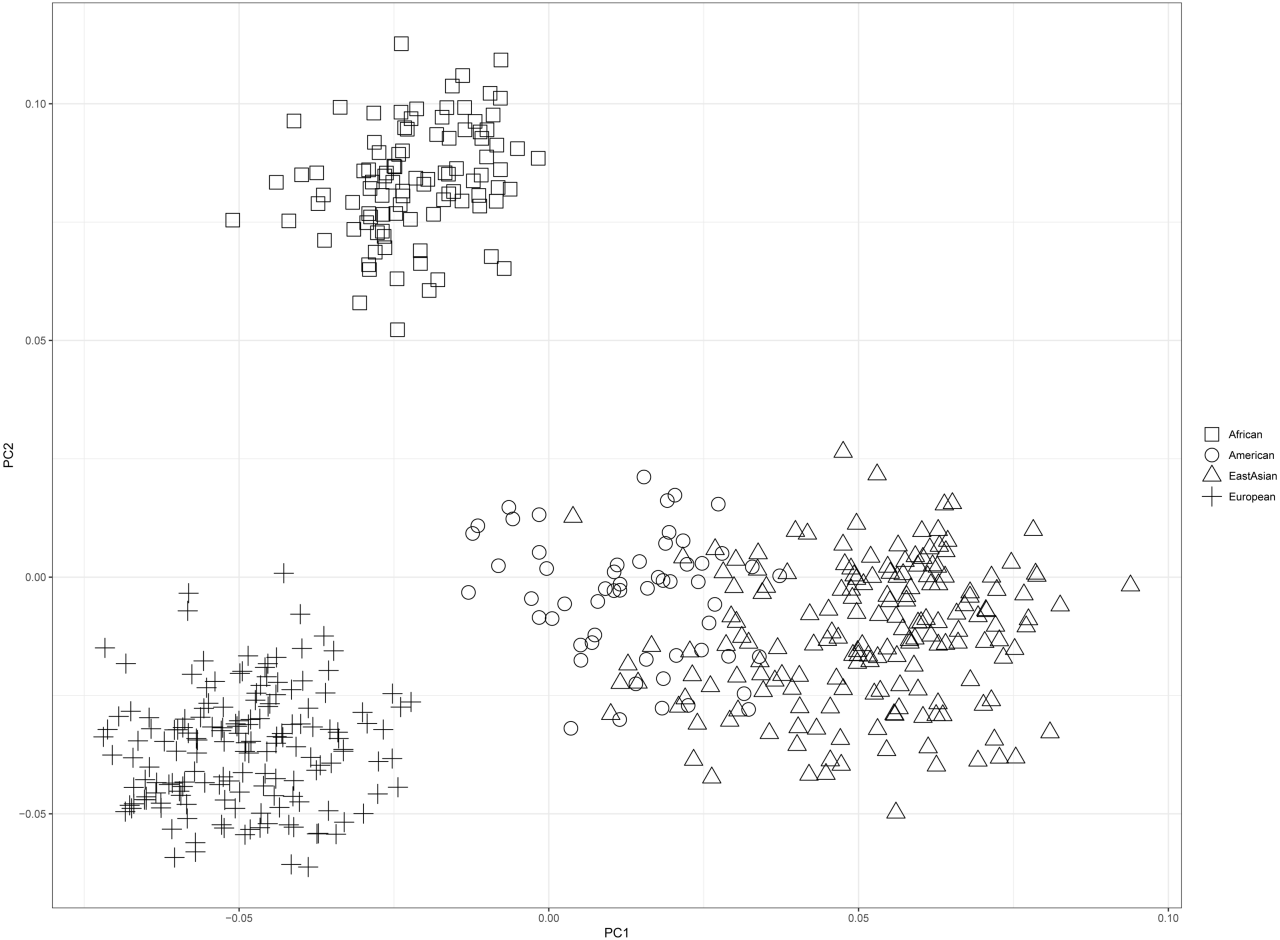
Principal component analysis of four continental populations comprising 25 training populations.

PCA of four continental populations comprising 25 training populations was shown in Fig. 3. Results revealed Africans and Europeans formed two distinct population clusters at quite some distances from the other populations. However, some American individuals clustered closely with East Asian individuals, which might be related to the shared ancestries before the divergences of East Asian populations and American populations (Li et al., 2008).

**Figure 4:**
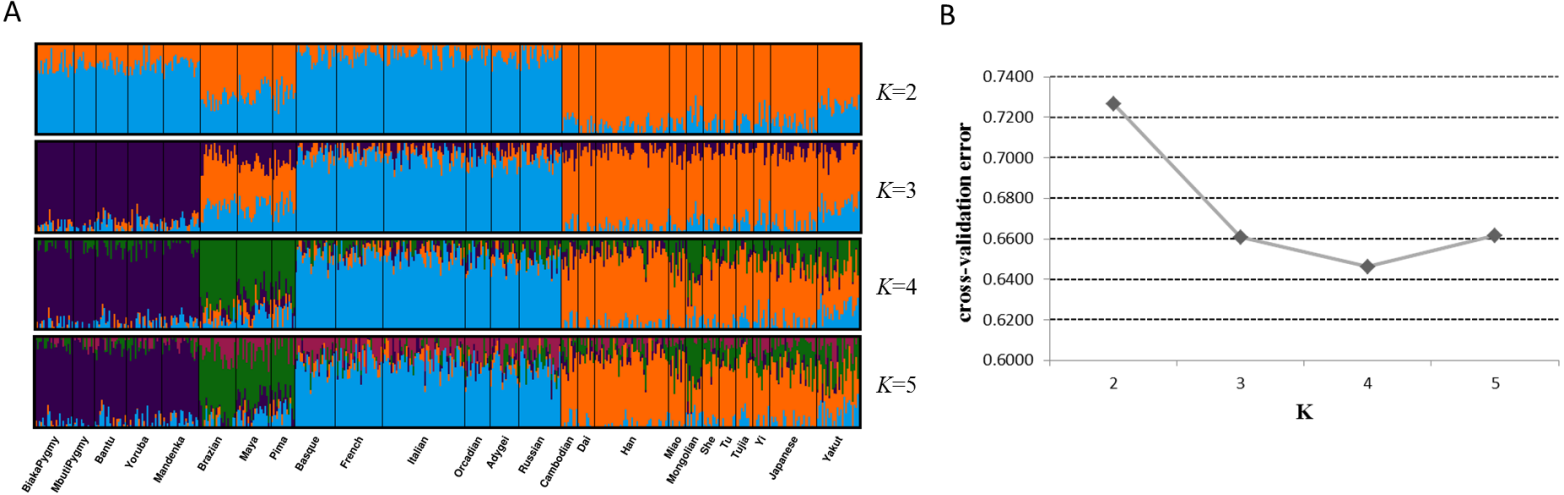
Genetic structure analyses of 25 training populations at *K* = 2–5 (A) and cross-validation error of each *K* value (B) based on 48 SNPs.

Next, we assessed genetic components of 25 training populations (Fig. 4A). At *K* = 2, the populations in Africa and Europe showed similar genetic components which could be distinguished from the populations in East Asia and America. At *K* = 3, African populations and European populations exhibited their distinct ancestry components, respectively. When *K* became 4, specific ancestry components in American populations could be observed and all training populations could be classified into four apparent clusters. No further distinctions among these populations could be discerned at *K* = 5. Given these results, apparent distinctions among these continental populations could be achieved by the 48 SNPs. Cross-validation error of each *K* value was estimated by ADMIXTURE software v1.3 to determine the optimum *K* value, as presented in Fig. 4B. Results revealed that the lowest cross-validation error was observed at *K* = 4, indicating *K* = 4 was the most appropriate for these data.

### Ancestry analysis of six testing populations based on 48 SNPs

**Figure 5:**
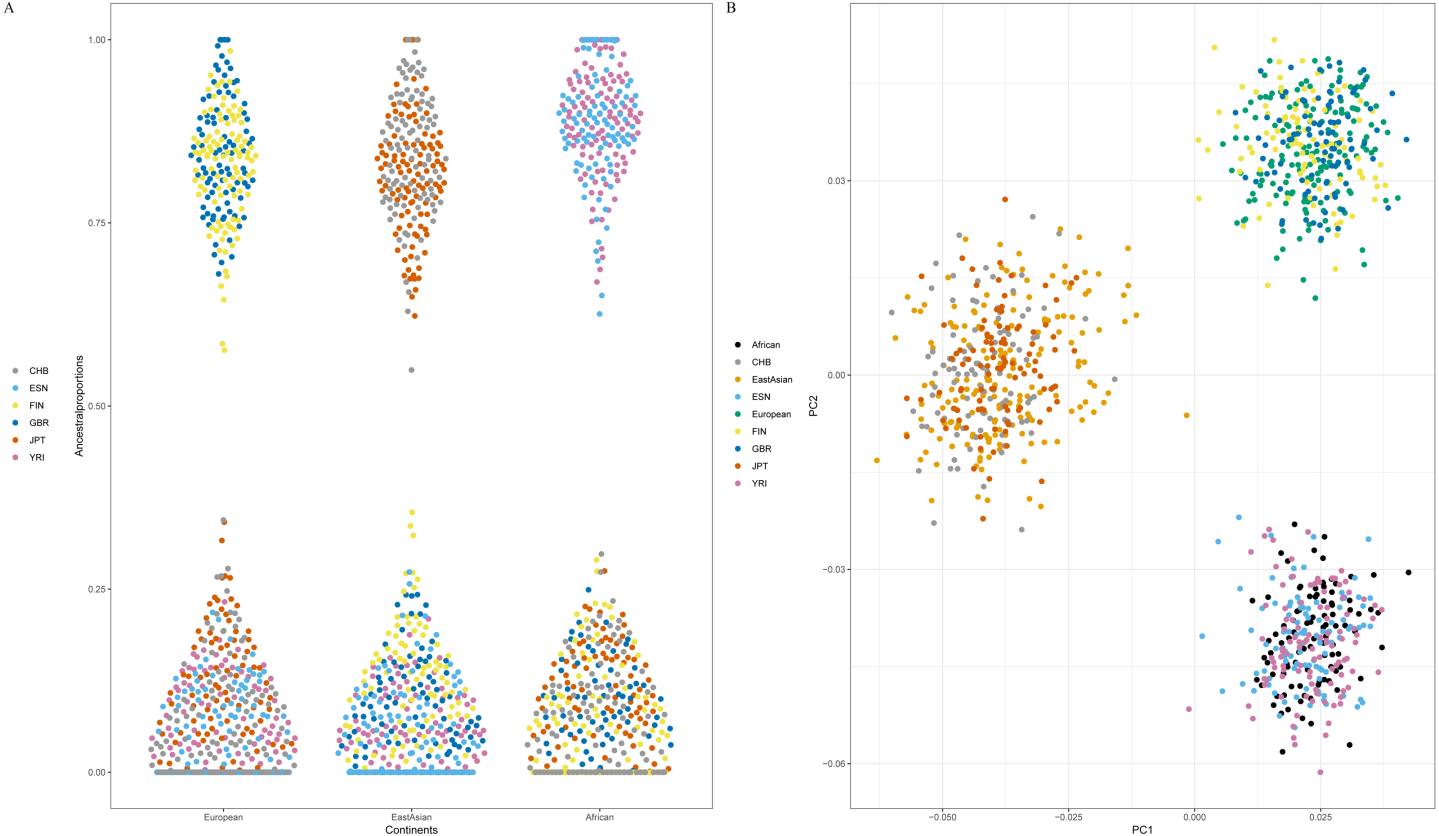
Ancestral origin analyses of six testing populations based on 48 SNPs. (A) genetic components of six testing populations by ADMIXTURE software v1.3. (B) Principal component analysis of six testing populations and three continental populations. Population abbreviations (CHB, ESN, FIN, GBR, JPT and YRI) are explained in Table 1.

Since American populations collected in 1000 Genomes Phase 3 showed different degrees of admixture components of European, American and African ancestries (Genomes Project Consortium et al., 2015), some populations from East Asia, Europe and Africa were employed to evaluate the efficiency of 48 SNPs for differentiating continental populations. The testing set included two African populations (ESN and YRI), two European populations (GBR and FIN) and two East Asian populations (CHB and JPT). Firstly, we estimated ancestry components of these six populations by ADMIXTURE software v1.3, as shown in Fig S1A. Results indicated that populations within the same continents showed similar genetic component distributions and could be separated from the populations in the other continents (Fig S1A). Furthermore, cross-validation plot revealed *K* = 3 was the best value for these testing populations (Fig S1B). Therefore, estimated genetic components of each individual for six testing populations at *K* = 3 were presented in Fig. 5A. We found FIN and GBR individuals showed high genetic components of European ancestry; CHB and JPT individuals possessed high genetic components of East Asian ancestry; ESN and YRI individuals demonstrated high genetic components of African ancestry. Consequently, these six testing populations could be assigned into their corresponding continental origins by these 48 SNPs.

Next, twenty-two training populations were classified into three known populations (African, European and East Asian) according to their biogeographical origins; six testing populations were treated as unknown individuals. PCA of these populations was conducted, as shown in Fig. 5B. Result demonstrated that African, European and East Asian individuals formed three population clusters. Moreover, we found that these testing individuals were superimposed onto the correct population clusters in Fig. 5B. Results of Naïve Bayes analysis also revealed all testing samples could be assigned to their corresponding continental regions (Table S4). For example, individual HG02922 was classified into African individuals with more than one billion times than European and East Asian individuals. From the above results, these 48 SNP set performed well for ancestry origin predictions of three continental populations (African, European and East Asian).

### Discrimination efficiencies of 48 SNP loci for three Chinese populations

**Figure 6:**
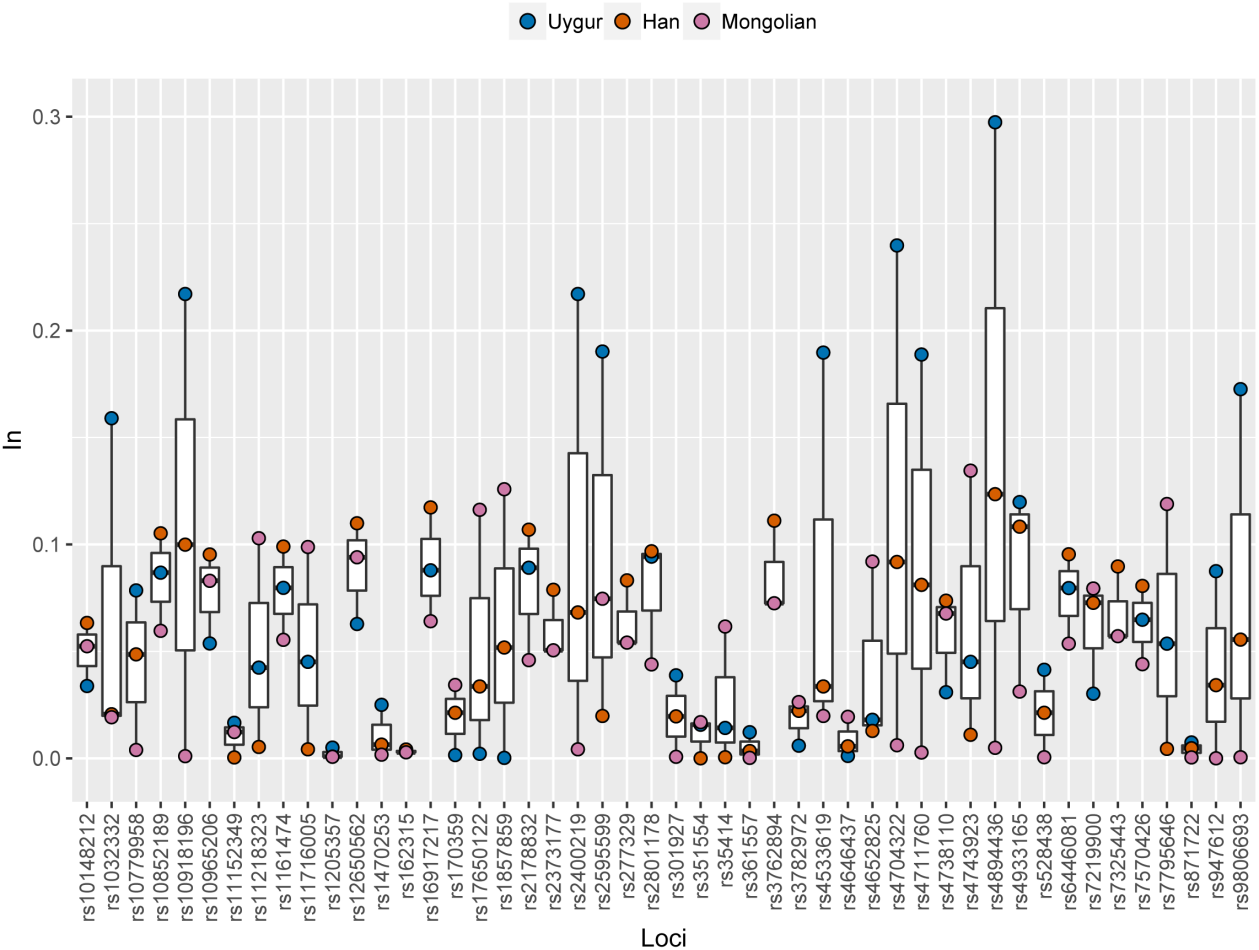
Population specific *In* values of 48 SNPs in Han, Uygur and Mongolian populations.

Ancestral allele frequencies of 48 SNP loci in Uygur, Han and Mongolian populations were given in Supplementary Fig. 2. Distinct frequency differences of most SNPs could be observed among pairwise populations. As an example, ancestral frequencies of rs1857859 locus in Uygur, Han and Mongolian populations were 0.65, 0.76 and 0.25, respectively, indicating the locus was good for distinguishing Mongolians from Uygurs and Hans. Population specific *In* values of 48 SNPs in these three Chinese populations were presented in Fig. 6 and Table S3. Results revealed that 10, 7 and 5 SNP loci displayed relatively high *In* values (≥0.1) in Uygur, Han and Mongolian populations, respectively. Additionally, some SNP loci which had low *In* values in four continental populations showed relatively high *In* values in one of the three Chinese populations. For example, rs10852189 locus whose *In* values in four continental populations were less than 0.05 had *In* value with being more than 0.1 in Han Chinese population.

**Figure 7:**
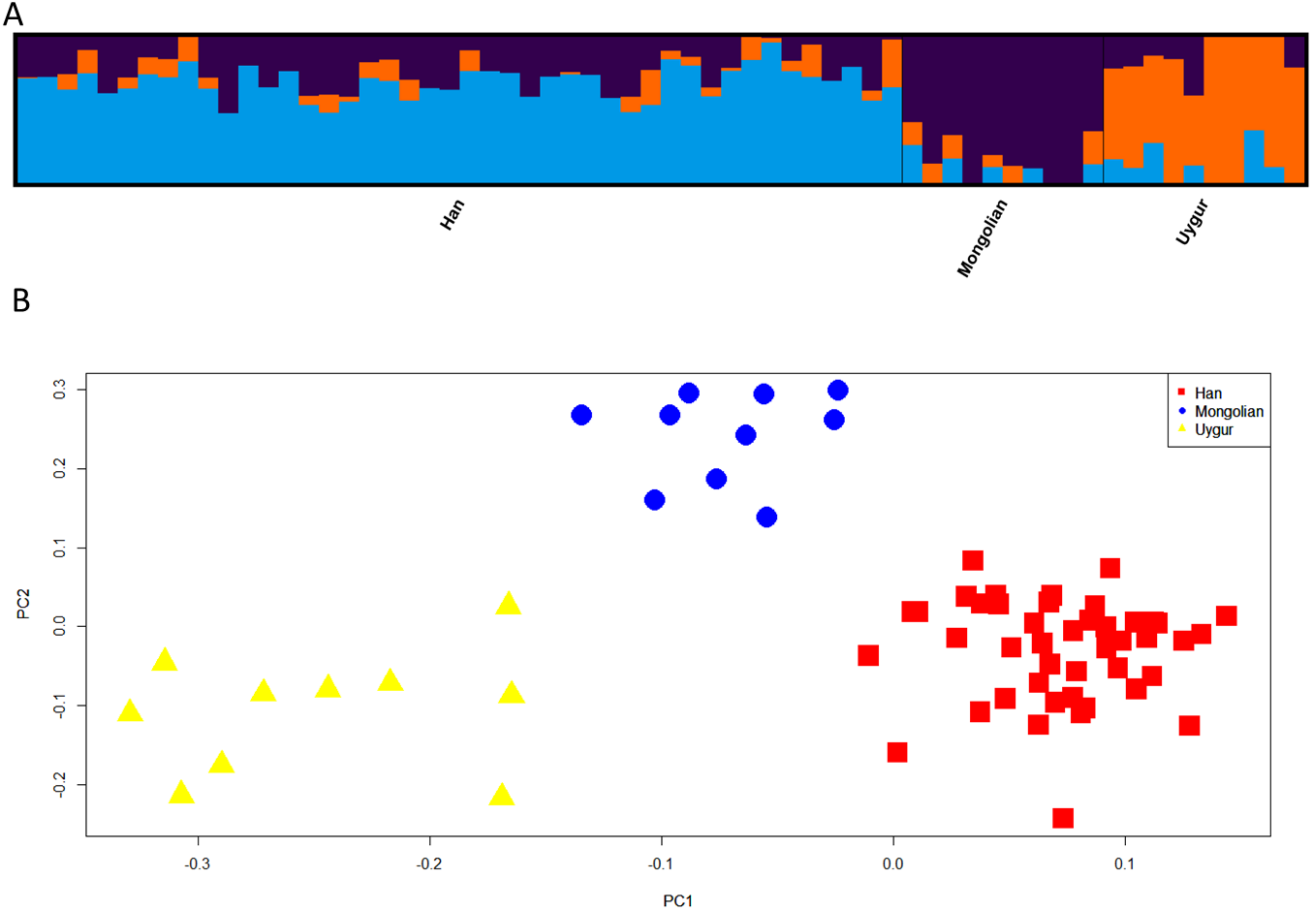
Genetic differentiation analyses among Han, Mongolian and Uygur populations in China. (A) Genetic structure analyses among these populations. (B) Principal component analysis of these populations.

Next, we assessed the power of 48 SNPs for ancestry analyses of Han, Mongolian and Uygur populations. Firstly, population structure analysis of three Chinese populations at *K* = 3 was given in Fig. 7A. Different ancestral component distributions were seen among these populations: Han population showed high blue proportions; Mongolian group displayed high purple proportions; Uygur group exhibited high orange proportions. We also found some individuals showed admixture ancestry proportions, which might result from the recent admixtures of these populations. Nevertheless, individuals from three Chinese populations formed three distinct clusters in the PCA plot (Fig. 7B). Moreover, multinomial logistic regression analyses of three Chinese populations were conducted to further evaluate the efficiencies of 48 SNP loci (Table S5). Results indicated Uygur, Han and Mongolian individuals were correctly classified into their corresponding populations with the probability values of 1.0000, reflecting these 48 SNP loci could estimate biogeographical origins of these Chinese populations well.

## Discussion

Ancestry origin predictions of different continental populations could be achieved by some SNP assays (Kidd et al., 2011; Kidd et al., 2014; Phillips et al., 2007). However, Soundararajan et al. (2016) stated that ancestral resolution among continental populations might be insufficient for the forensic application. Therefore, several SNP panels which were used to improve the ancestral resolution of regional populations have been developed by some researchers (Bulbul et al., 2018; Li et al., 2016; Phillips et al., 2013). In the current study, we presented one set of SNPs which were utilized to differentiate different continental populations and three Chinese populations.

For continental populations (African, American, East Asian and European), we found these 48 SNPs could perform well for ancestry analyses of individuals from Africa, East Asia and Europe although some SNPs were not informative in these continental populations. Moreover, some American individuals were observed to be overlapped onto East Asian individual cluster (Fig. 3). Nonetheless, distinct genetic components among these populations were discerned from Fig. 4A. To obtain better ancestral resolutions among Americans and other continental populations, more highly differentiated SNPs in American populations should be selected. What’s more, future research should be paid more attention to ancestry inferences of within-continental populations. The 1000 Genomes Project has assessed genetic variations of 2,504 individuals from five continents and found that some variants were unique to some populations (Genomes Project Consortium et al., 2015). Therefore, the variations private to one population should be selected to enhance resolution power among within-continental populations.

For Uygur, Han and Mongolian populations, previous research investigated their genetic variations based on different genetic markers (Mei et al., 2016; Tao et al., 2018). Tao et al. (2018) assessed genetic polymorphisms of 12X-STRs and found that the Mongolian group showed indistinguishable genetic component distributions when compared to the components of Han populations in different regions. Mei et al. (2016) conducted genetic differentiation analyses between Uygur and other reference populations based on 30 InDels and found that Uygur group was far from Han populations and other Chinese populations in the PCA plot. In this study, we selected 48 SNPs to differentiate Han, Mongolian and Uygur populations. Compared with the study for the identification of Japanese people, Yuasa et al. (2018) selected the SNPs with the Japanese-specific alleles to differentiate Japanese individuals from the other East Asian populations. The similar method could be employed to select the SNPs with population-specific alleles so as to obtain better ancestral distinctions among different ethnic groups in China. Moreover, some research concerning on genomic analysis of a great many of Chinese individuals has been reported (Chiang et al., 2018; Liu et al., 2018). We will make full use of these data to further screen those highly differentiated genetic markers in different ethnic groups in China in the future.

Although these 48 SNP loci perform well for ancestry origin inferences of three continental populations (African, East Asian and European) and three Chinese populations (Han, Mongolian and Uygur), some loci of the 48 SNPs who had lower *In* values were not suggested to perform ancestry analyses of these populations so that we could obtain more accurate results for ancestry origin predictions. For example, SNPs whose *In* values in continental populations were less than 0.1 should not be employed to infer biogeographical origins of continental populations given that high genetic differentiations among different continental populations existed; SNPs whose *In* values in subpopulations from the same regions were less than 0.05 should not be utilized to perform ancestry analysis among these populations. Furthermore, there were some limitations in the current study. On one hand, the validation of 48 SNP loci in new samples was not conducted, especially for Han, Mongolian and Uygur populations. On the other hand, sample sizes of Uygur (10), Han (44) and Mongolian (10) were relatively small. Accordingly, it is necessary for us to further validate the efficiency of these 48 SNP loci for ancestry analysis in larger samples.

## Conclusions

To conclude, forty-eight SNPs were provided to differentiate different continental populations, which could provide valuable clues for forensic investigations. Furthermore, these SNPs could also be utilized to differentiate three populations residing in China, which achieved fine-scale resolutions in regional populations. Further validation for these 48 SNPs should be conducted in the large sample set. What’s more, the SNPs with population-specific alleles should be screened to obtain better ancestral resolutions among regional populations.

##  Supplemental Information

10.7717/peerj.6508/supp-1 Table S1 The *P*-values of Hardy–Weinberg equilibrium tests of 48 SNP loci in 25 training populations by Genepop software v4.0 Note. (A), Bantu population includes Kenya Bantu and South African Bantu populations. (B) Brazian population includes Karitiana and Surui populations. (C) Italian population includes Sardinian, Tuscan and Bergamo populations. ’-’ means that *P*-value of Hardy–Weinberg equilibrium of the locus cann’t be estimated in training populations by Genepop software v4.0 because only one allele is presented, or two alleles are detected but one is represented by only one copy.

10.7717/peerj.6508/supp-2 Table S2 Population specific *In* values of 48 SNPs in four continentical populations and three Chinese populations Note: (A) Population specific *In* values in four continental populations were calculated based on the genetic data of 25 training populations. (B) Population specific In values in Han, Mongolian and Uygur populations were calculated based on the genetic data of these three populations.

10.7717/peerj.6508/supp-3 Table S3 Naïve Bayes analysis of six testing populations and three continental populations (African, European and East Asian) Note. Three continental populations consists of 22 training populations (excluding from American populations) in Table 1. Values are the number of individuals who are classified as continental populations. Numbers in parentheses are the percentages of testing individuals who are classified as continental populations.

10.7717/peerj.6508/supp-4 Table S4 Multinomial logistic regression analysis of three Chinese populations based on 48 SNP loci

10.7717/peerj.6508/supp-5 Table S5 Genetic data of 48 SNPs in populations used in this study The training set and three Chinese populations were downloaded from CEPH-HGDP (Li et al., 2008). The testing set was downloaded from 1000 Genomes Phase 3 (Genomes Project Consortium et al., 2015).

10.7717/peerj.6508/supp-6 Figure S1 Genetic structure analyses of six testing populations at *K* = 2–5 (A) and cross-validation error of each *K* value by Admixture software v1.3 based on 48 SNPs Population abbreviations are explained in Table 1.

10.7717/peerj.6508/supp-7 Figure S2 Ancestral allele frequencies heatmap of 48 SNPs in Han, Mongolian and Uygur populations from China Different colors represent for different the levels of frequency values: pink for low value; green for high value.

## Additional Information and Declarations

### Data Availability

The following information was supplied regarding data availability: Genetic data of 48 SNPs used in this study were given in Table S1.
