# Supplementary figures and images for: A set of novel SNP loci for differentiating continental populations and three Chinese populations
Source: PeerJ. 2019 Mar 29;7:e6508. doi: 10.7717/peerj.6508 (PMC6445247; doi:10.7717/peerj.6508)

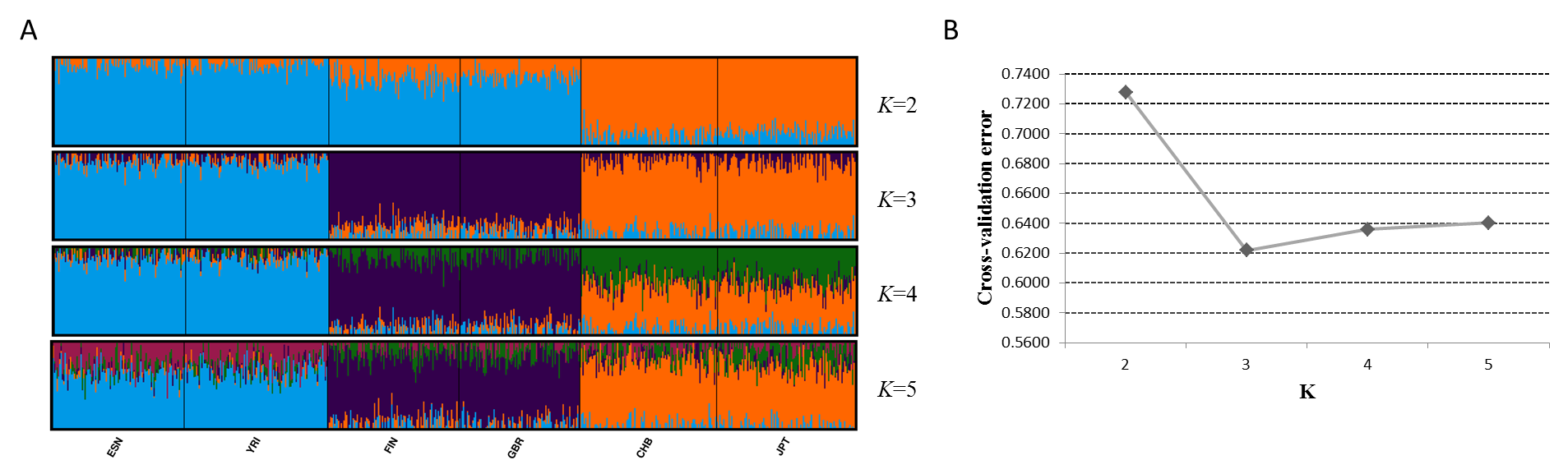

Supplement: Figure S1 — Population abbreviations are explained in Table 1. [file peerj-07-6508-s006.png]

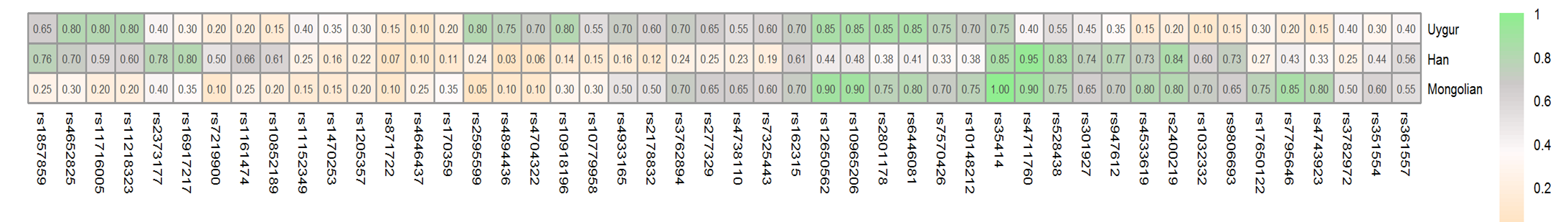

Supplement: Figure S2 — Different colors represent for different the levels of frequency values: pink for low value; green for high value. [file peerj-07-6508-s007.png]
